# Fascioliasis in Llama, *Lama glama*, in Andean Endemic Areas: Experimental Transmission Capacity by the High Altitude Snail Vector *Galba truncatula* and Epidemiological Analysis of Its Reservoir Role

**DOI:** 10.3390/ani11092693

**Published:** 2021-09-14

**Authors:** Santiago Mas-Coma, Maria Mercedes Cafrune, Ilra Renata Funatsu, Atilio Jose Mangold, Rene Angles, Paola Buchon, Maria Cecilia Fantozzi, Patricio Artigas, Maria Adela Valero, Maria Dolores Bargues

**Affiliations:** 1 Departamento de Parasitologia, Facultad de Farmacia, Universidad de Valencia, Av. Vicent Andres Estelles s/n, 46100 Burjassot, Valencia, Spain; Ilra.Funatsu@uv.es (I.R.F.); cfantozzi@fcv.unl.edu.ar (M.C.F.); Patricio.Artigas@uv.es (P.A.); Madela.Valero@uv.es (M.A.V.); 2 Instituto de Investigación Animal del Chaco Semiárido, Área de Investigación en Salud Animal, Estación Experimental Agropecuaria Salta, Instituto Nacional de Tecnología Agropecuaria (INTA), Ministerio de Agricultura, Ganadería y Pesca CIAP, Ruta Nacional 68–km 172, Cerrillos A4403, Salta, Argentina; cafrune.wierna@inta.gob.ar; 3 Estación Experimental Agropecuaria Rafaela, Instituto Nacional de Tecnologia Agropecuaria (INTA), Ministerio de Agricultura, Ganadería y Pesca, CC 22 INTA Rafaela, Rafaela 2300, Santa Fe, Argentina; mangold.atilio@inta.gob.ar; 4 Cátedra de Parasitología, Facultad de Medicina, Universidad Mayor de San Andrés (UMSA), Av. Saavedra, Miraflores, La Paz, Bolivia; anglesrene@hotmail.es; 5 Unidad de Limnología, Instituto de Ecología, Universidad Mayor de San Andrés (UMSA), Campus Calle 27, Cota Cota, La Paz, Bolivia; pbuchon31@gmail.com

**Keywords:** Andean fascioliasis endemic areas, llama, *Fasciola hepatica*, *Galba truncatula*, experimental transmission, field epidemiology, reservoir role, Argentina, Bolivia

## Abstract

**Simple Summary:**

The infection by the liver fluke *Fasciola hepatica* in South American camelids, mainly llamas and alpacas, has been the focus of many studies. However, their capacity to participate in the transmission of the disease and their potential reservoir role in human or animal endemic areas have never been studied. Therefore, all development stages of an isolate from Argentine llama of the high Andean plateau have been experimentally investigated, from egg embryogenesis to metacercarial infectivity, by using the vector snail *Galba truncatula* from the high altitude Bolivian Altiplano human hyperendemic area. Although eggs shed by llamas may successfully develop until the adult stage in a subsequent mammal host, the transmission capacity of the llama proved to be pronouncedly less efficient than that of other hosts as sheep and cattle. Moreover, the low prevalences, intensities, and daily fecal outputs of liver fluke eggs in llama in Andean endemic areas, together with their peculiar defecating behavior in dung piles always far from freshwater collections, indicate that the contribution of this camelid should be considered negligible. Therefore, the llama does not need to receive priority within fascioliasis control initiatives, although it may play a disease-spreading role if used as a pack animal.

**Abstract:**

South American camelids are definitive hosts of *Fasciola hepatica*. However, their capacity to participate in the transmission and epidemiology of fascioliasis has never been appropriately studied. Therefore, an *F. hepatica* isolate from Argentine llama is for the first time analyzed using *Galba truncatula* lymnaeids from Bolivia. Experimental follow-up studies included egg embryogenesis, miracidial infection of lymnaeid snails, intramolluscan larval development, cercarial production, chronobiology of cercarial shedding, vector survival to infection, and metacercarial infectivity of mammal host. Shorter prepatent and patent periods were leading to markedly lower cercarial production, shorter cercarial shedding, and a higher negative impact on snail survival. The usually low liver fluke prevalences and intensities and low daily fecal outputs indicate that llamas do not substantially contribute to fascioliasis transmission. The defecating behavior in dung piles far from freshwater collections prevents lymnaeid infection by eggs shed by this camelid. All results suggest the reservoir role of the llama to be negligible and, therefore, no priority within control measures in endemic areas. However, llamas may play a disease-spreading role if used as pack animals in rural areas. In the Northern Bolivian Altiplano human hyperendemic area, neither llamas nor alpacas should be considered for control measures within a One Health action.

## 1. Introduction

The two trematode species *Fasciola hepatica* and *F. gigantica* are transmitted by freshwater gastropod snails of the family Lymnaeidae and cause a parasitic disease affecting domestic and sylvatic mammals, as well as humans. Fascioliasis is a highly pathogenic disease with a large economic impact on husbandry of livestock in different countries [1,2] as well as globally, including countries where human infection by these fasciolids is a wide health problem [3]. An estimation of total world economic losses in only cattle and buffalo reached USD 3.2 billion [4].

In humans, fascioliasis may also be highly pathogenic and include severe clinical pictures [5,6,7] and sequelae [5,8]. In areas where it is endemic in humans, it may cause community underdevelopment [9,10], as is the case of many rural areas of developing countries where the immune suppression it causes [11] underlies very frequent coinfections with other pathogenic parasites [12,13] and microorganisms [14,15]. Moreover, the transmission and epidemiology of this disease have proved to be strongly influenced by climate change [16,17] and also by global change aspects such as anthropogenic modifications of the environment, man-made movements of domestic ruminants, and livestock importation and exportation between countries, and even continents [18].

The description of several human endemic areas in different continents, where infection may occur very early in life [19], and the increasing number of reports on fasciolid infected patients throughout, not only in low-income countries but also in developed countries [20], led the World Health Organization to include this disease among the group of food-borne trematodiases within the list of Neglected Tropical Diseases (NTDs) [21]. Within this framework, the WHO launched a worldwide strategy of preventive chemotherapy based on the availability of a very efficient drug for human treatment, triclabendazole [22]. The programs of control were designed according to the different human endemic countries depending on the characteristics of their patterns of transmission and their epidemiological scenarios. Strengthening these programs is key in the new NTD road map 2030 of the WHO, in the way for the sustainability of the health systems [23].

In South America, *F. hepatica* is the causal agent of many human fascioliasis endemic areas, which are mainly distributed in altiplanos and valleys at high altitudes in the Andean region. In these areas, the aforementioned preventive chemotherapy strategy is now being complemented by One Health interventions to decrease the risks of infection and reinfection. Control measures implemented within these interventions include the assessment of the contributions of the different livestock reservoir species to the transmission and epidemiology of the disease [24,25,26], among many other action axes [27,28,29].

The present study focuses on the potential role of South American camelids in the transmission and epidemiology of the disease in the very high altitude Andean endemic area. Indeed, infection by *F. hepatica* has been reported in the four species of South American camelids, namely the domesticated llama (*Lama glama*) and alpaca (*Lama pacos*), and the sylvatic but also semi-captive vicuña (*Vicugna vicugna*) and guanaco (*Lama guanicoe*). However, their capacity to transmit fascioliasis has never been evaluated.

These camelids grow in herds throughout the Andean mountains in altitudes higher than 2500 m where they are well adapted to the extreme conditions of this environment. The husbandry of llamas and alpacas has been traditionally implemented for meat and milk production, as well as for their use as pack animals in rural areas. More recently, their interest has been increased in the fur industry because of the quality of their wool and the countries have therefore launched initiatives to improve their husbandry, among which control measures against their infectious diseases [30].

The aforementioned reports in South American camelids concern the Andean countries where the most public health important human fascioliasis endemic areas are known to be present, mainly in high or very high altitude areas:

- in Argentina, there is the risk of human infection in several provinces and human endemic areas that has been described [31,32,33]; *F. hepatica* infection reports in South American camelids of this country concern llama [34], vicuña [35,36], and guanaco [37,38];
- in Chile, *F. hepatica* infected patients have been repeatedly reported from different political regions, including areas presenting human endemicity [39,40]; llamas and alpacas have been reported to be infected by the liver fluke in this country [41];
- in Bolivia, the human fascioliasis hyperendemic area where the highest prevalences (up to 72% by coprology and 100% by serology) and intensities (up to 8,000 eggs per gram of feces—epg—in children) have been reported in the Northern Bolivian Altiplano, at 3800–4100 m a.s.l., in between Lake Titicaca and the city of El Alto and the capital La Paz [12,42,43]; *F. hepatica* infection has been reported in alpacas raised on a farm of Belen, northward of the human endemic area [44,45];
- in Peru, human fascioliasis endemic areas have been described throughout many valleys along the whole north-south distributed Andean chain [13,46,47,48,49,50,51,52,53] and also in the Peruvian Altiplano [54]; the camelid species having been found infected by the liver fluke in Peru include the llama [55,56], alpaca [56,57,58,59], and vicuña [60,61].

Most of these reports on liver fluke infection of camelids concern animal endemic areas, but the reports in the Mantaro valley [56] and in the province of Huancavelica [60] refer to human endemic areas in Peru [46,47,49]. The report in the Northern Bolivian Altiplano refers to a farm located close to the human hyperendemic area [44,45].

In trematodiases, for a host species to act as a reservoir of the disease the capacity of the adult parasite to develop in this definitive host is not sufficient. It should be experimentally verified that this host participates in the transmission of the parasite. For such a purpose, the whole life cycle of the parasite through and after the involvement of this host should be followed up until the successful infection of a subsequent definitive host. Moreover, surveys in the endemic area should demonstrate that prevalence and intensity rates in this host species are sufficient to assure a stable transmission in the field. For this assessment of transmission capacity and epidemiological involvement in South American camelids, the llama was chosen for the present study. A llama isolate from the Argentine high Andean plateau and the snail vector *Galba truncatula* from the hyperendemic area of human fascioliasis in the Northern Altiplano of Bolivia were used.

The laboratory studies described herein included the experimental follow-up of all the life cycle phases, comprising the complete development of the egg and miracidium of an *F. hepatica* isolate obtained in infected llamas from the high Andean plateau of Argentina, the infection of Bolivian *G. truncatula* vector snail specimens, the development of the larval stages of the liver fluke in this snail up to final production of cercariae, the shedding of cercariae and their chronobiological features, the effects of the infection on the life span of the vector snails, and finally, the infection capacity of the metacercariae in a laboratory model host.

To assess the participation of the llama in the transmission of *F. hepatica* and its contribution to the epidemiology of the disease, the results obtained in the laboratory and in the field are quantitatively compared with the results previously obtained in the *F. hepatica* isolates from the two main reservoir species sheep and cattle in the same hyperendemic area of Bolivia [24]. To allow for the obtaining of significant results, the experimental studies undertaken included exactly the same aspects and were made according to identical methods and techniques. Field surveys to assess prevalences, intensities, and egg outputs further help in evaluating the potential role of the host species as a reservoir of the disease. Such complete experimental and field studies focus on a South American camelid species for the first time.

**Figure 1:**
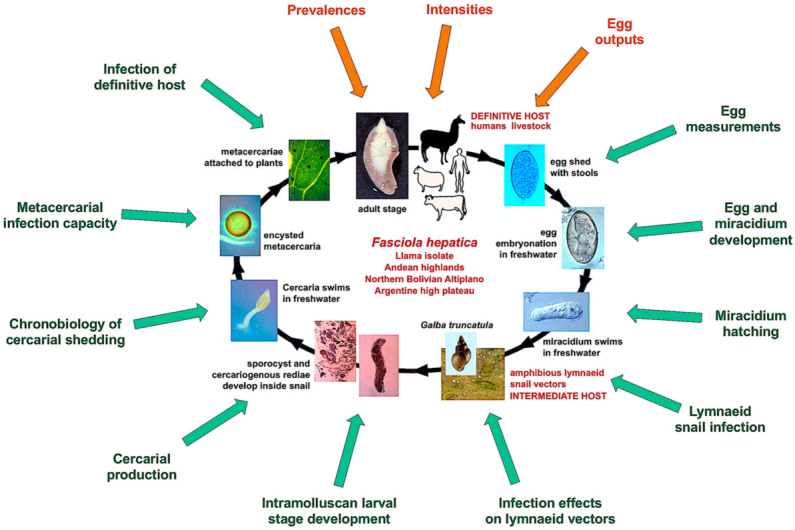
Life cycle of *Fasciola hepatica* including detailed development phases: Aspects assessed for the characterization of the transmission capacity and epidemiological role of the llama from Andean highlands by comparing with sheep and cattle isolates from the Northern Bolivian Altiplano human hyperendemic area. Green arrows: experimental assessments; brown arrows: field assessments. Schematic drawing by S. Mas-Coma.

A detailed illustration of the complete two-host life cycle of *F. hepatica* is included in Figure 1, showing all the aforementioned aspects assessed for the characterization of the transmission capacity and epidemiological role of the llama from Andean highlands, by comparing with sheep and cattle isolates from the Northern Bolivian Altiplano human hyperendemic high altitude area.

## 2. Materials and Methods

### 2.1. Fasciolid Materials

**Figure 2:**
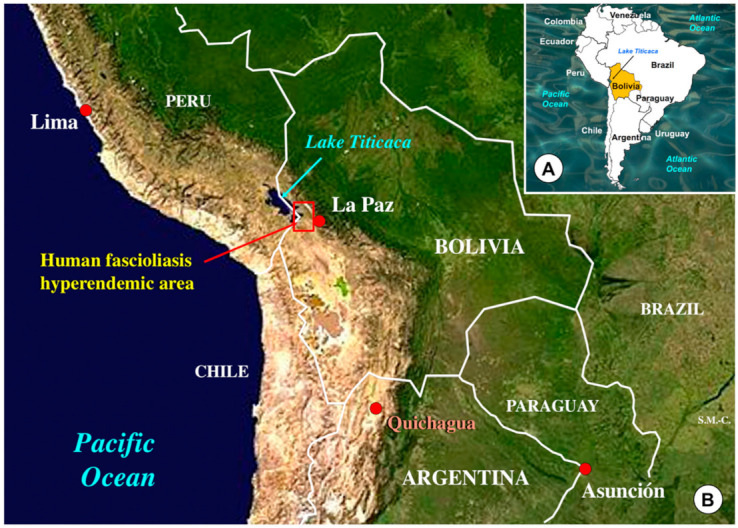
Map showing the locality of Quichagua in the Andean high plateau, at 3586 m altitude in the North of Argentina where *Fasciola hepatica* materials were collected from llamas, and the human fascioliasis hyperendemic area, in the Northern Altiplano of Bolivia, at 3820–4100 m altitude, where lymnaeid snail vector specimens of *Galba truncatula* were collected. Background for (**B**) from composed satellite map of South America orthographic projection by NASA (full resolution of 1215 Å ~ 1712 pixels; public domain) via Wikimedia Commons. (**A**,**B**), originals S. Mas-Coma.

Original *F. hepatica* materials were obtained from livers, bile aspirates, and fecal samples of naturally infected llamas in an area close to the locality of Quichagua (22°48′00.72″ S; 66°01′59.97″ O; altitude of 3586 m), located in the Department of Cochinoca, province of Jujuy, Argentine high Andean plateau (Figure 2). This area is an animal fascioliasis endemic area where different species of livestock are infected by the liver fluke [62].

Fluke eggs of llama isolate were obtained from fecal and bile samples. These *F. hepatica* eggs were recovered after isolation by filtration and subsequently shortly maintained in natural water. A temperature of 4 °C and total darkness were applied until the moment for launching the assessment of the embryogenesis of the eggs.

Lymnaeid snails of the species *G. truncatula* from the endemic area of the Northern Altiplano were used for the snail vector experimental assays. *Fasciola hepatica* eggs from llamas were also isolated by filtration of fecal samples and similarly kept until used to obtain miracidia for the experimental infection of the snails.

The Wistar rat served as a laboratory model host to assess the infection of the definitive host. Wistar rat infection was performed with metacercariae of the *F. hepatica* isolate of the llama. These metacercariae came from the previous infections of the Altiplanic lymnaeid snails and were maintained in natural water at a temperature of 4 °C in complete darkness. These are standard conditions in experimental work for studies on fasciolid flukes [63].

### 2.2. Embryogenesis of Fluke Eggs

The embryogenesis of eggs was separately studied in eggs collected from feces and in eggs found in bile, following the same procedure. In both cases, the embryogenesis was microscopically followed at a constant temperature of 20 °C and at time intervals of four days. The different phases of the development of the eggs were considered to assess the embryogenesis, including (i) eggs including a morula, with visible vitelline granules and/or spheroidal cells (E.M.), (ii) eggs including an outlined miracidium, i.e., a miracidial pre-form can already be visualized (E.O.M.), and (iii) eggs including a developed miracidium, i.e., a well defined, completely conformed miracidium is visible (E.D.M). In addition to the eggs in the stages of E.M., E.O.M., and E.D.M., (iv) degenerated eggs (DE), (v) empty eggs (EE), and (vi) broken eggs (BE) were also considered and counted. A total of 33 eggs from each host individual were counted in each 4-day study under the microscope between the slip and coverslip, by distinguishing each egg in which of the aforementioned six phases it was. Appropriate images to illustrate these six stages of the fasciolid eggs have been previously published [25]. Egg counts were noted in percentages independently per each day of observation. For the evaluation of the embryogenesis process, comparisons with the egg embryogenesis curves of the sheep and cattle isolates of *F. hepatica* from the Northern Bolivian Altiplano human hyperendemic area, obtained following the same procedures and from a similar very high altitude [24], were performed.

### 2.3. Experimental Infection of Snails

The lymnaeid species *Galba truncatula*, a member of the *Galba*/*Fossaria* group and considered the most efficient liver fluke vector species [64,65], was selected for the experimental procedures. Laboratory-borne snails derived from *G. truncatula* population specimens collected in the locality of Ancocagua, at 3,841 m a.s.l., in the Pucarani municipality of the Northern Bolivian Altiplano human hyperendemic area (Figure 2), were used. The genetic characteristics and systematic classification of this population have been previously assessed [28]. Living specimens collected in Ancocagua were transported under isothermal conditions for their laboratory adaptation to standardized controlled conditions of 20 °C, 90% relative humidity (r.h.), and a 12 h/12 h light/darkness photoperiod in precision climatic chambers (HPS-1500, VB-0714 and HPS-500 models of Heraeus-Vötsch, Calservice Heratec, S.L., Madrid, Spain).

Before the adaptation of the snails to the laboratory, a possible infection by liver flukes acquired in nature was discarded. For this purpose, each snail was maintained in isolation in a small amount of natural water inside a small Petri dish. The presence or absence of moving cercariae or motionless cysts of metacercariae was looked for in each Petri dish under a binocular microscope after a period of 24 h. The non-infected snails were distributed in standard breeding boxes containing 2000 mL natural freshwater, which was weekly changed. Lettuce and algae were provided as food ad libitum.

Hatching of developed miracidia was forced by putting fully embryonated eggs under light and the miracidia obtained were afterwards used for the experimental infection of snails [66]. The infection susceptibility was assessed in a total of 45 lymnaeid snails of a size of 4.0–5.0 mm. Each snail was exposed to one miracidium for 4 h in a small Petri dish containing 2 mL of fresh water. The disappearance of the miracidium was taken as verification of its successful penetration into the snail. The mono-miracidial infection with the *F. hepatica* isolate from llama was carried out under the experimental conditions of 20 °C/20 °C day/night temperature according to a photoperiod of 12 h/12 h light/darkness in the aforementioned climatic chambers [67]. For the evaluation of the snail infectivity of the llama isolate, comparisons with the results of the sheep and cattle isolates of *F. hepatica* from the Northern Bolivian Altiplano human hyperendemic area, obtained following the same procedures with the same lymnaeid vector species of the same endemic area and from a similar very high altitude [24], were performed. Characteristics, conditions, and snail number in these infection experiments, together with the aforementioned comparisons, were appropriately noted.

Once the experimental infection was performed, the lymnaeids were again transferred to the 2000 mL containers and maintained at a permanent temperature of 20 °C, at a relative humidity of 90%, and a light/darkness photoperiod of 12 h/12 h day/night. Dry lettuce ad libitum was used as food for 30 days. At 30-days post-infection (d.p.i.), the lymnaeid specimens were again individually put in isolation for the daily follow-up of the shedding of cercariae. In each Petri dish, the metacercariae were counted daily for the chronobiological assessment of the cercarial shedding. For both periods of shedding and post-shedding, lettuce was furnished ad libitum to each lymnaeid inside a dish until lymnaeid death.

### 2.4. Laboratory Infections of Wistar Rats

Wistar rats (Iffa Credo, Barcelona, Spain) were used to assess the definitive host infection capacity of the llama *F. hepatica* isolate. A total of 25 Wistar male rats, 4–5 weeks of age, were used as a laboratory mammal model. Previously reported standards were strictly followed, including a commercial diet for these animals (Panlab Chow A04) and natural water ad libitum [68].

The experimental infection of the aforementioned Wistar rats was carried out as previously reported [69]. A total of 20 metacercariae was the dose applied for the experimental infection of each rat. A gastric tube was used for the inoculation. Special attention was given to the care and health of the animals. Rat weight and fur appearance were checked weekly in a way to assess the body condition and well-being of the rats by comparing with negative controls. At the end of the experiment, a lower weight was presented by the infected rats. None of the rats died. A necropsy performed at 12 weeks post-infection was undertaken to establish the percentage of rats infected and the intensity or burden concerning the number of flukes obtained in each infected animal. An overdose of a selected anesthetic (IsoFlo; Dr. Esteve SA, Barcelona, Spain) was finally applied to humanely euthanize the animals. The search and collection of flukes were performed by means of a dissecting microscope, following a methodology previously described [15,70]. Flukes were searched first in the bile duct and afterwards throughout the whole liver. All other organs were also analyzed, especially both abdominal and thoracic viscera and cavities, by thoroughly rinsing with water to recover all flukes.

For the evaluation of the definitive host infectivity of the llama isolate, comparisons with the results of the sheep and cattle isolates of *F. hepatica* from the Northern Bolivian Altiplano human hyperendemic area, obtained with metacercariae recovered following the same procedures with the same lymnaeid vector species of the same endemic area and from a similar very high altitude [24], were performed.

### 2.5. Field Surveys of Llamas

A total of 841 llamas could be coprologically diagnosed, namely 811 llamas from Argentina including 103 from the high Andean plateau of Quichagua and 708 from the provinces of Salta, Jujuy, and Catamarca, and 30 llamas from Bolivia, in the locality of Palcoco in the Northern Altiplano (Figure 2). Fresh fecal samples were immediately placed in a plastic bag, transported to the laboratory within the following 5 h, and maintained at 4 °C until examination.

Fecal samples from Argentina were processed by a modified technique of flotation [71,72]. The analysis of the samples from Bolivia was performed by means of a modified sedimentation test [73,74]. In the samples of both geographical origins, an amount of 4 g of fecal sample was used to allow for the quantitative analysis. The number of eggs shed by a llama was used to estimate the infection intensity and was expressed in eggs per gram of stools (epg). A host individual was considered negative when no eggs were found in its respective fecal sample after studying three slides.

In Quichagua, the availability of an occasionally slaughtered llama female was additionally used to analyze the liver infection by fasciolids and to further obtain samples of bile aspirates.

Fasciolid eggs found in both feces and bile were used for their size assessment. The fasciolid eggs were measured by means of a computer image analysis system (CIAS). Standardized measurements used were those which have already proved their usefulness in fasciolid trematodes [75]. These measurements were obtained with a digital 3CCD color video camera (Sony DXC-930P, Sony España, S.A., Barcelona, Spain) adapted to a microscope. The captured images were analyzed by means of an image analysis software (ImagePro plus version 5.0 for Windows, Media Cybernetics, Rockville, MD, USA). The characteristics of the fasciolid eggs which were studied comprised the following values: (a) linear measurements including the egg length (EL), egg width (EW), and egg perimeter (EPe); (b) areas including the egg area (EA); and (c) ratios including the EL/EW ratio. The mean and standard deviation plus minimum and maximum values were determined for each measure.

### 2.6. Statistical Analyses

To quantify the participation of the llama to the *F. hepatica* transmission and to evaluate the reservoir role of this camelid, the results obtained in each aspect were appropriately compared with the same results obtained with *F. hepatica* isolates of sheep and cattle from the hyperendemic area of human fascioliasis in the Northern Altiplano of Bolivia. These two ruminant species have previously been demonstrated to be the main transmitters and reservoirs of the disease in that high altitude area. To assure the significance of these comparisons, the methods and techniques used for the analyses of the Altiplanic sheep and cattle isolates were exactly the same as those used for the llama isolate, regarding experiments in the laboratory as well as surveys in the field [24].

Statistical analyses were performed by applying the SPSS Statistics 26 software. The ANOVA test was employed for the analysis of the data of E.M., E.O.M., E.D.M. in the embryogenesis of the fasciolid eggs. Both the Chi-square test and Yates continuity corrected Chi-square test were utilized for the comparisons of categorical variables. The non-parametric Kruskal–Wallis test was applied for the comparisons of mean data obtained in the experimental infections of both lymnaeid vectors and laboratory rats. The post hoc tests (L.S.D., Student–Newman–Keuls and Duncan’s tests) were used for the analyses of the size variable comparisons concerning EL, EW, EPe, EA, and EL/EW of the fasciolid eggs. In all analyses, the results were considered statistically significant when *p* < 0.05.

## 3. Results

### 3.1. Egg Embryonation

**Figure 3:**
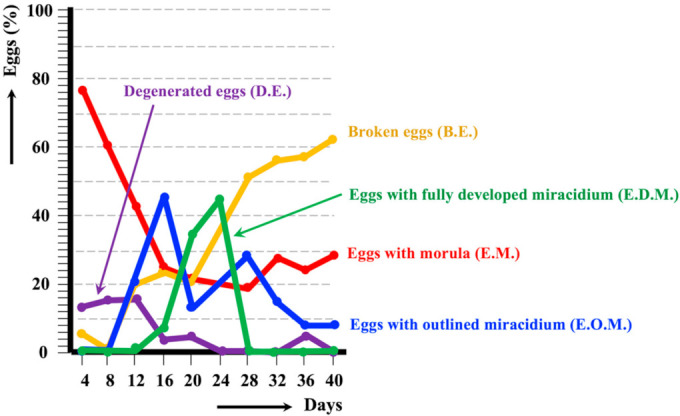
Graph showing the results of the experimental follow-up study of the egg embryonation of the isolate of *Fasciola hepatica* obtained from llama feces, and analyzed at 4-day intervals and constant temperature of 20 °C.

The embryonation of *F. hepatica* eggs collected from fecal samples from llama was followed at 20 °C up to the final miracidium development (Figure 3). The study showed that eggs including an outlined miracidium (E.O.M.) appeared from day 12, whereas eggs containing a completely conformed miracidium (E.D.M.) began to be detected from day 16. These E.D.M. eggs were subsequently found in each microscopic observation between days 16 and 24. E.D.M. egg percentages proved to follow a curve increasing up to a peak surpassing the 40% on day 24, after which it rapidly decreased to 0 on day 28, indicating that eggs including an outlined miracidium after this day were unable to subsequently give rise to a fully developed miracidium. The progressive increase in the percentage of broken eggs from day 12 up to day 40 is worth noting, similarly as the relatively high percentage of almost 30% of eggs still showing an inner morula on day 40. The average egg embryonation (E.O.M., E.D.M.) along the 40 days showed lower values in the llama fecal isolate (20.14%) when compared to the sheep isolate (45.16%) and cattle isolate (29.78%) from the Altiplano (*p* < 0.05).

**Figure 4:**
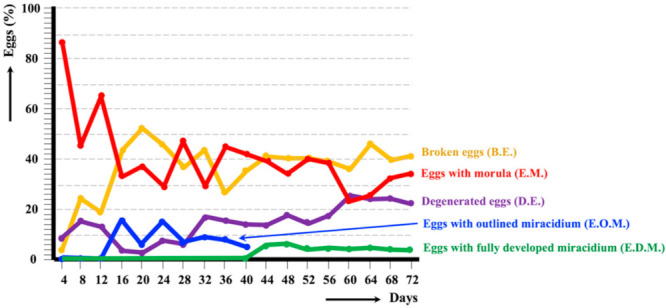
Graph showing the results of the experimental follow-up study of the egg embryonation of the isolate of *Fasciola hepatica* obtained from llama bile aspirate, and analyzed at 4-day intervals and constant temperature of 20 °C.

In eggs collected from bile, embryonation could also be experimentally followed at 20 °C up to the total conformation of the miracidium (Figure 4). The observational analyses showed that E.O.M. eggs appeared from day 16, but the first E.D.M. eggs only appeared from day 44 onwards. Eggs containing a fully developed miracidium were continuously observed daily until day 72, although always in percentages lower than 10%. The relatively high percentages of broken eggs and degenerated eggs, as well as eggs still presenting a morula at day 72, should be highlighted. The average egg embryonation (E.O.M., E.D.M.) along the 72 days showed lower values in the llama bile isolate (18.36%) when compared to the sheep isolate (38.46%) and cattle isolate (26.63%) from the Altiplano (*p* < 0.05).

### 3.2. Snail Infectivity and Intramolluscan Development

The results of the snail vector infection assays performed with the Argentine llama isolate, including their comparison with the altiplanic *F. hepatica* isolates from sheep and cattle, are shown in Table 1. The llama isolate (33.3%) was demonstrated to be somewhat more efficient than the isolate from cattle (25.0%). Both llama and cattle isolates proved to be pronouncedly less efficient than the isolate from sheep (51.8%). However, no statistically significant differences in the percentage of lymnaeids successfully infected by a mono-miracidial dose at 20 °C/20 °C (=the snail vector infectivity) between the llama isolate and the isolates of two ruminant reservoirs in the Bolivian Altiplano (*p* > 0.05) were detected.

The prepatent period of the llama isolate (39.4 days) showed an evidently lower mean value than the isolates from altiplanic sheep (55.6 days) and cattle (55.5 days). These differences in the prepatent period between the llama isolate and the isolates from the two main reservoirs proved to be statistically significant (*p* < 0.05). The period of cercarial emergence in the isolate of llama showed a mean value which proved to be pronouncedly shorter (mean: 12.2 days) than in those in the isolates from sheep (mean: 34.7 days) and cattle (mean: 47.1 days). From the statistical point of view, the duration of this emergence of cercariae in the llama isolate was significantly different from the length of this period in the isolates from sheep and cattle (*p* < 0.05).

The comparison of the production of cercariae between these *F. hepatica* isolates also furnished significant differences. The statistical analyses demonstrated that the cercarial production considered for each infected lymnaeid in the isolate from llama (mean: 51.3 cercariae/lymnaeid) was significantly different from those in the isolate from sheep (mean: 197.9 cercariae/lymnaeid) and cattle (mean: 306 cercariae/lymnaeid) (*p* < 0.05).

Interesting results were obtained in the analysis of the influence of the infection on the lymnaeid vectors, including the aspects of the survival of the lymnaeids after the end of shedding days, the longevity of the infected shedding lymnaeids, and also the survival length of the non-shedding lymnaeids. In the isolate from llama, all of the aforementioned aspects were demonstrated to be statistically different from those in the isolates from sheep and cattle (*p* < 0.05). These differences were clearly obvious by both the mean and maximum dpi values, which were markedly shorter in the llama isolate when compared to the isolates from altiplanic sheep and cattle (Table 1).

### 3.3. Chronobiological Pattern of The Cercarial Emergence

**Figure 5:**
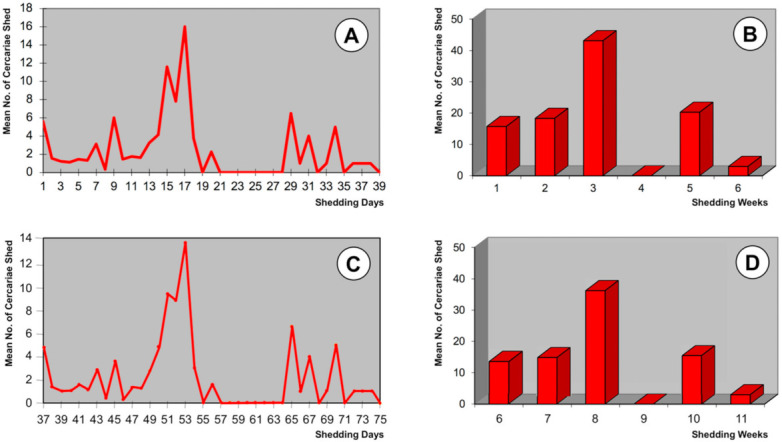
Chronobiological patterns of cercarial emergence by *Galba truncatula* from the Northern Bolivian Altiplano monomiracidially infected with the llama isolate of *Fasciola hepatica* from the Argentine high Andean plateau: (**A**,**B**) shedding period analyzed according to the mean amounts of cercariae shed daily and weekly from the day of the emergence of the first cercaria by each snail; (**C**,**D**) shedding period analyzed according to the mean amounts of cercariae shed daily and weekly from the day of the miracidial infection; prepatent period not shown.

The chronobiology of the cercarial shedding observed in the llama isolate is shown in Figure 5. The shedding period was analyzed according to two points of view (Figure 5A–D).

On one side, the study concerned the average amounts of cercariae which were shed daily (Figure 5A) and weekly (Figure 5B) by all shedding lymnaeids involved in a follow-up from the day of the emergence of the first cercaria by each snail. The complete length of this period lasted up to 38 days or 6 weeks, with an average of 12.2 days (Table 1). The daily process of cercarial emergence showed several waves according to an irregular succession including several peaks. Among them, the highest peak does not appear until day 17, i.e., pronouncedly delayed regarding the first cercarial shedding day. The curve computing weekly emergences showed that it was throughout the third week when cercariae shed presented their highest numbers. Interestingly, there is a period of lack of daily shedding between days 21 and 28, after which shedding restarts and is irregularly maintained until final exhaustion.

Conversely, the emergence was studied from the day of the miracidial infection of each snail. In this second analysis, the period of the emergence of cercariae showed daily and weekly curves (Figure 5C,D) which are very similar to those of the chronobiology when analyzed from the day of the first cercarial shedding by each lymnaeid (Figure 5A,B). This is due to the fact that 70% of the snails, including the big shedders (up to 92 cercariae/snail), started shedding on the same day, i.e., identical prepatent period, whereas the remaining 30% delayed snails were only specimens that shed a small number of cercariae (8–23 cercariae/snail; mean 14.3 cercariae/snail).

### 3.4. Experimental Infectivity of Mammal Host

Assays performed in the laboratory to assess the infection capacity of metacercariae of the Argentine *F. hepatica* isolate from llama and carried out with Wistar rats are shown in Table 2. The comparison of infectivity results concerning the percentages of successfully infected animals (52.0% in the llama isolate versus 78.4%; and 75.0% in the sheep and cattle isolates, respectively) demonstrated that, despite the evidently lower value in the llama isolate, there were no statistically significant differences (*p* > 0.05). Additionally, the infection burden or intensity, expressed by fluke number per inoculated metacercariae and per animal, neither showed significant differences in the comparison of the isolate from llama with that from sheep (*p* > 0.05).

### 3.5. Prevalence, Intensity, Egg Measurements and Shedding Rates

A prevalence of 21.6% was found in a study of fecal samples from 88 llamas of 39 herds in the Argentine high Andean plateau of Quichagua area, but a pronouncedly lower prevalence of 4.2% was later observed in fecal samples from 708 llamas of 89 herds coming from the Argentine provinces of Salta, Jujuy, and Catamarca.

In Quichagua, infection intensity could be assessed in 14 female llamas and 1 male llama aged 2 years by fasciolid egg counting in their fecal samples. The quantitative coprological analyses showed an intensity ranging between 1 and 10 epg. Additionally, a total of 62 adult flukes could be recovered from the liver of an occasionally slaughtered female llama, which presented with macroscopic hepatic lesions suggesting chronic infection despite no visible symptom attributable to liver fluke infection.

**Figure 6:**
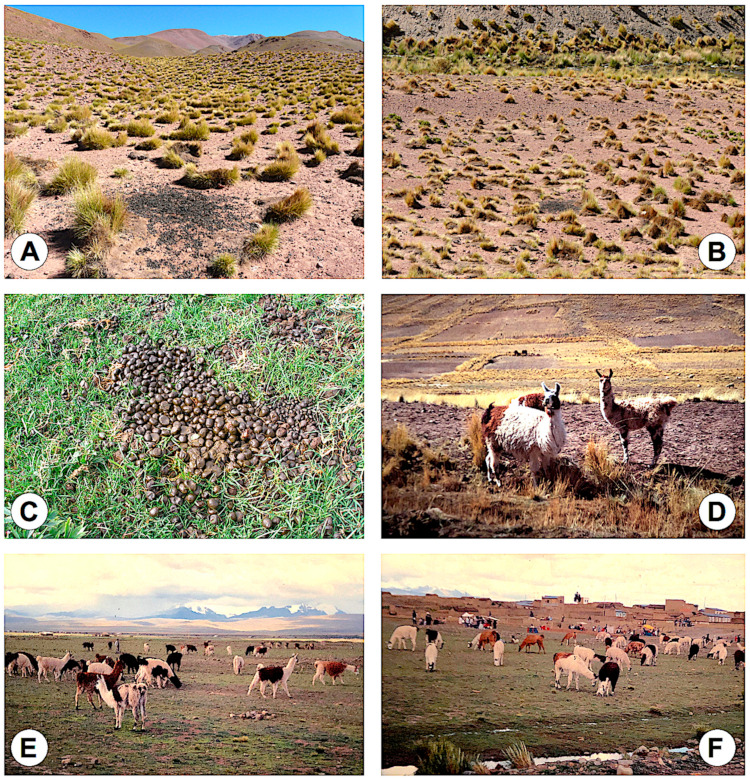
Llamas in the Argentine high Andean plateau and the Northern Bolivian Altiplano: (**A**) Typical dung pile in an Argentine Andean highland prairie; (**B**) Another dung pile located far away from small stream that rises from the slope sources in an Argentine Andean highland prairie (see background); (**C**) Dung pile showing fecal particles of llama commonly called “llama beans”; (**D**) Llamas in the northeastern part of the “corridor of Peñas” (see slope of the mountainous foothills of the Eastern Andean chain in the background and note surrounding aridity and absence of freshwater collections); (**E**,**F**) weekly trade fair of llamas in the locality of Palcoco in the Northern Bolivian Altiplano (note the absence of big “llamas cargueras”). (**A**,**B**): Photographs by A.J. Mangold; (**C**): Photograph by M.M. Cafrune; (**D**–**F**): Photographs by S. Mas-Coma.

In the Northern Bolivian Altiplano, fecal samples from llamas could only be obtained at a weekly trade fair of llamas in the locality of Palcoco (Figure 6E,F) in the month of March, which is usually the last month of the rainy season. A total of 30 llamas present in the Palcoco trade fair were surveyed. None showed liver fluke eggs in their feces.

**Figure 7:**
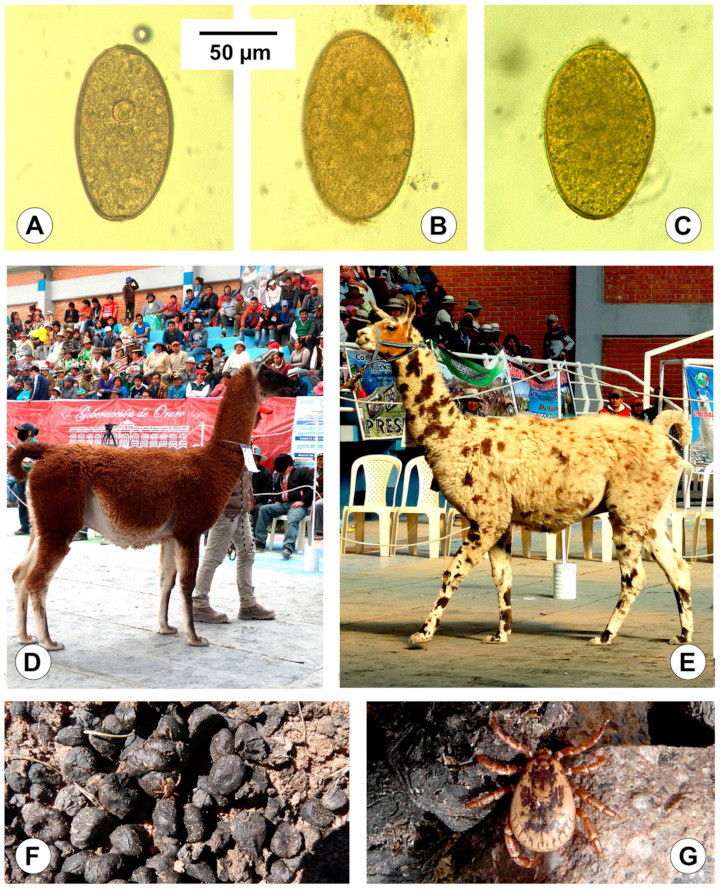
Fascioliasis and llamas in the high Andean plateaus of Argentina and Bolivia: (**A**–**C**) Eggs of *Fasciola hepatica* in fecal samples of llamas from Quichagua, Argentina (photographs at the same scale); (**D**,**E**) llamas for good transport (“llamas cargueras”, “Karas” or “Peladas”) in the yearly national camelid trade fair in the locality of Turco, Oruro, in the Central Bolivian Altiplano (note their big size when comparing with the height of the person behind); (**F**,**G**) Tick specimens of *Amblyomma parvitarsum* waiting in between “llama beans” (this specific tick behavior indicates a very long evolutionary adaptation to a particular defecating behavior of its llama host). (**A**–**C**): Photographs by M.M. Cafrune; (**D**,**E**): Photographs by Ing. Hugo Eduardo Lamas (Abra Pampa, Jujuy, Argentina); (**F**,**G**) Photographs by A.J. Mangold.

A total of 36 and 37 eggs from fecal and bile samples of llama, respectively, were measured (Figure 7A–C). Table 3 includes the measurements obtained in the morphometric study of the *F. hepatica* eggs of the llama isolate and their comparison with the same measurements of eggs from sheep and cattle of the Bolivian Altiplano. The corresponding analyses by means of post hoc statistical tests, such as L.S.D., Student–Newman–Keuls, and Duncan’s tests, showed that the liver fluke eggs in llamas are significantly smaller than the eggs in the ruminant isolates, except the length/width ratio (*p* < 0.05).

When considering the infection intensity in llamas varying between 1 and 10 epg and the number of stools defecated by a llama individual per day, the total number of *F. hepatica* eggs fecally shed by a camelid individual per day could be calculated (Table 3). The daily egg output per llama per day proved to be pronouncedly lower than the same estimations made for both sheep and cattle.

## 4. Discussion

### 4.1. Egg Embryonation

#### 4.1.1. Egg Embryonation in the Llama Isolate

In the life cycle of liver flukes belonging to the genus *Fasciola*, eggs fecally shed by several species or geographical strains of local hosts are known to be unable to reach the total embryonation of the miracidium. The development of the miracidium inside the egg is thus a crucial characteristic to look for when assessing the viability of a mammal species isolate. Indeed, egg formation, production, development, and viability are aspects analyzed within the egg hatch assays used for drug efficiency evaluation [78].

Egg embryonation is a temperature-dependent process. The time needed to reach a fully developed miracidium inside the egg is longer at lower temperatures. A temperature of 20 °C has been used for the egg embryonation of the llama isolate to allow for the appropriate evaluation by comparison with the sheep and cattle isolates also from very high altitude and analyzed at the same constant temperature according to exactly the same methodology to obtain significant results.

In ruminant isolates, the hatching time at 20 °C has been observed to vary between 19–20 days [79] and 27 days [80]. In the isolates of sheep and cattle isolates from the Northern Altiplano of Bolivia, the first completely conformed miracidium appeared at day 24, and eggs presenting this final miracidium stage were uninterruptedly found until the end of the observational study of 143 days [24]. In the llama isolate, the stage of fully developed miracidium was reached in both follow-up studies of eggs collected from fecal samples and from bile. However, embryonation results in both feces and bile indicate an egg development pronouncedly less successful than in the sheep and cattle isolates. The high percentage of eggs including a morula throughout the whole follow-up shows that many eggs are not able to continue their development. Moreover, the high percentage of broken eggs, whether gradually increasing in feces (Figure 3) or maintained at high values in bile, together with the increasing proportion of degenerated eggs also in bile (Figure 4), indicate a lack of viability in many eggs.

#### 4.1.2. Miracidial Infectivity, Intramolluscan Development and Cercarial Chronobiology

There is a large complexity and variability in the processes of snail infectivity and fasciolid larval stage development linked to the many different factors involved, as has been observed in studies about the interactions between *F. hepatica* and *G. truncatula* [81].

The snail infectivity of the llama isolate with a mono-miracidial dose at a constant temperature of 20 °C was 33.3% (Table 1). This is in agreement with the 14.0–56.8% range experimentally found in different populations of the same lymnaeid vector species *G. truncatula* in France [82,83], similarly as regarding the 25.0–51.8% observed in altiplanic isolates from sheep and cattle [24].

The prepatent period (from infection up to the shedding of the first cercaria) in the llama isolate proved to be shorter than that found in altiplanic sheep and cattle isolates [24] (Table 1), which in their turn proved to be slightly longer than in *F. hepatica*/*G. truncatula* of the lowlands [79,84,85]. This shorter prepatent period of the llama isolate remembers the similar precociousness observed in the embryogenesis of viable eggs from llama.

The mean length of the patent period (length of the cercarial shedding period) in the llama isolate also proved to be pronouncedly shorter than the same period in the sheep and cattle isolates from the Bolivian Altiplano [24] (Table 1) and also from that in *F. hepatica* from ruminants of the lowlands [82,86].

The cercarial shedding chronobiology in the llama isolate shows similar daily and weekly patterns in both the curve from the day of the emergence of the first cercaria by each snail (Figure 5A,B) and in the curve from the day of the miracidial infection (Figure 5C,D), because most infected snails began simultaneously with shedding. In all these curves, two shedding phases separated by a non-shedding period in week four are observed. Instead of an initial shedding peak and subsequent gradual progressive decrease as observed in the altiplanic sheep and cattle isolates [87], in the llama isolate there was only a delayed acrophase. The pattern of shedding of cercariae found in the llama isolate does neither fit the pattern of 1–14-waves, including the pattern of 4–5-waves followed by the majority, observed in *F. hepatica* infecting *G. truncatula* of the lowlands analyzed under constant temperature and photoperiod conditions [88]. The pauses in the curve analyzed from the day of the miracidial infection have been linked to the redial generation replication processes [87]. In the case of the llama isolate, results may be interpreted as including only two cercariogenous redial generations, one before and the other after the aforementioned multiday pause.

#### 4.1.3. Cercarial Production, Lymnaeid Survival and Metacercarial Infectivity

Worth mentioning is the cercarial production capacity of the llama isolate, which was 51.3 cercariae/snail, a markedly lower quantity when compared to the 197.9 cercariae/snail and 306.0 cercariae/snail of the sheep and cattle isolates from the Bolivian Altiplano, respectively [24] (Table 1). This llama isolate production is even lower than the low productions found in *F. hepatica* infecting *G. truncatula* in several populations of lowland areas, such as 91.7 cercariae/snail [89] or 120.0 cercariae/snail [90].

Snail survival after shedding end, the longevity of shedding snails, and the longevity of non-shedding snails in infections by the llama isolate are also shorter than in those obtained when infecting with the altiplanic sheep and cattle isolates (Table 1) [24]. Interestingly, this longevity has been proven to be longer in infected snails inhabiting areas of high altitude than the duration of the same period in *F. hepatica* infecting *G. truncatula* in the lowlands, a phenomenon which has been observed to be independent on the host isolate of *F. hepatica* [91].

The results of the experimental infections of Wistar rats showed the definitive host infectivity of metacercariae of the llama isolate (Table 2). The experimental prevalence obtained fitted well in the results obtained with the altiplanic sheep and cattle isolates, and the intensity with that of the sheep isolate [24], and also in the present knowledge when dealing with short-aged metacercariae of ruminant origin [63,92].

### 4.2. Epidemiological Role of the Llama

A prevalence of 21.6% was found in the animal endemic Argentina high Andean plateau at Quichagua and a prevalence of 4.2% in subsequent wider surveys in neighboring zones in Argentina. However, none of the 30 llamas analyzed in the Northern Bolivian Altiplano at Palcoco showed infection. Indeed, local prevalence of liver fluke infection in llamas varies pronouncedly according to localities: between 8% and 35% in Peru [55,93], 16.4% in the USA [94], 49.5% also in Peru [56], and up to 80% in Argentina [34,95,96].

In the Northern Bolivian Altiplano human hyperendemic area, llamas can only very sporadically be observed. Besides the few specimens present in the garden of the Titicaca Hotel and the Tiwanaku ruins, both for mere touristic purposes, only a few individuals can regularly be found in the north-eastern part of the altiplanic corridor of Peñas (Figure 6D) and in the altiplanic corridor of Huancarani-Jesús de Machaca. However, the latter two places lie outside of the wide human endemic area. Additionally, llamas concentrate once a week in a trade fair specialized on this Andean camelid in the locality of Palcoco. The llamas of this weekly fair mainly come from zones outside of the endemic region, such as from:

- the zone located north of the endemic area, at a higher altitude on the way to the Eastern Andean Chain; it should be considered that there is no fascioliasis transmission in places located at an altitude higher than 4000 m a.s.l. due to the inability of the transmitting lymnaeids to survive at the temperature of such extreme altitudes [28];
- the altiplanic zone located south of the endemic area, on the way to Oruro, where temperatures are colder owing to the absence of the milder climatic influence of Lake Titicaca [97], which similarly explain the absence of lymnaeid vectors [28]; indeed, none among a total of 404 llamas from the Oruro department (82.4% from Sajama province, 8.7% from San Pedro de Totora province, 5.4% from Carangas province, and 3.5% from Litoral province) analyzed in the slaughterhouse of the locality of Turco, close to the city of Oruro, were found to be infected [98].

Liver fluke infection has been reported from alpacas in the Northern Bolivian Altiplano. However, it should be highlighted that, in the Northern Bolivian Altiplano, besides a very few isolated individuals together with llamas regularly found in the northeastern part of the “corridor of Peñas” (Figure 6D), alpacas are only found on the farm of Belén, northward of Achacachi, both outside of the human fascioliasis transmission area. Previous coprological studies on alpacas from Belén farm [44,45] reported the following results: in one study 59.1% of 22 alpacas were detected shedding *F. hepatica* eggs, with averages of 75, 16, and 2 eggs found in animals with high (n = 7), moderate (n = 2) and low (n = 2) infection, respectively; in another survey, 25 alpacas among 29 studied showed eggs in their feces (86.2%), of which 22 alpacas presented fewer than 10 epg, 2 alpacas showed between 10 and 30 epg, and only 1 alpaca presented more than 30 epg. According to these data, alpacas may be taken into account in the zone of the Belén farm but can be discarded regarding a potential reservoir role in the human endemic area.

The intensity of 1–10 epg found in llamas from Quichagua is very low when compared with the intensities found in altiplanic sheep and cattle (Table 3). In llama, egg output ranges reaching pronouncedly higher maximums have been reported, such as 0.6–253.2 epg in domesticated specimens in the USA [99] and 1–332 epg (mean 42 epg) in a farm in the UK [100], although lower ones of 12.6 ± 1.9 epg (geometric mean ± standard deviation) were found in endemic areas of Peru [56]. The 62 liver flukes recovered from the slaughtered llama in Quichagua is a number lower than the 154 flukes found in experimentally infected llamas [99].

*Fasciola hepatica* eggs shed by naturally infected llamas from Quichagua showed size and form characteristics similar to those previously found in this camelid species: 123.2/68.6 μm in the USA [99]; 102.1–148.4/57.9–86.9 μm (mean 126.7/72.0 μm) with an EL/EW ratio of 1.76 in Peru [56]. The measurements of liver fluke eggs shed by llamas prove to be smaller than those shed by sheep and cattle isolates from the Northern Bolivian Altiplano (Table 3) [24,101].

The total number of liver fluke eggs daily expelled with feces by a llama in Quichagua, according to the number of stools defecated by a llama per day [76,77], may be estimated to be between 700 and 39,000 eggs/llama/day (Table 3). Only slightly higher rates around 49,140 eggs/llama/day are obtained when considering egg outputs by llamas in endemic areas of Peru [56]. Such ranges appear to be markedly lower than those calculated for the main reservoirs of sheep and cattle [24].

Llamas appear to be very susceptible to fascioliasis, with a histopathological picture similar to that shown by sheep [99]. The animal survival to infection should be taken into account in control initiatives, mainly in areas of high transmission rates leading to very high individual fluke intensities by infection and re-infection [102,103], such as in the Northern Altiplano human hyperendemic areas of Bolivia [24] and Peru [54], or in Andean valleys of Peru [13]. It should be considered that the contribution to the transmission of fascioliasis may only be for a short time in the case of a mammal that only survives a given period after the liver fluke infection. Such a contribution may therefore be negligible when compared with other definitive hosts which are able to transmit for longer periods or even the post-infection rest of their long life [25].

The more common hosts of *F. hepatica* may be divided into three groups based on an early, delayed, or low level of resistance [104]:

- Group I: It includes early resistance hosts characterized by possessing tissues that are not suitable for the fluke and resulting in a high degree of natural resistance. The infection is self-limiting without harming the host. The domestic pig is an example.
- Group II: This concerns the delayed resistance hosts characterized by a resistance that is acquired during the first weeks of a primary infection or during challenge infection. A delayed host reaction controls flukes during tissue migration, and chronic reactions including bile duct calcification lead to the eventual elimination of infection. Mortality is not common. Cattle and horses represent this group.
- Group III: Host species of this group have low resistance resulting in severe tissue reactions that do not immobilize or eliminate the parasites. In the chronic condition, there is no calcification of the bile ducts and flukes often survive the life of the host. Mortality in both the acute and chronic phases is common. Sheep and goats are hosts included in this group.

The bile ducts of South American camelids show more similarity with the equine bile system than with the bile system of domestic ruminants [105]. Despite this, the histopathological picture of fascioliasis in llamas suggests a low resistance to liver fluke infection and has been reported to be more similar to that in sheep [99,100,106] than to that in equines [107]. Indeed, *F. hepatica* infection is a common cause of production loss and mortality in domesticated New World camelids [44,55,57,58,108]. Its impact in alpacas has led to many studies about diagnostics and treatment [58,59,109].

Nevertheless, surveys in Andean endemic areas showed low intensities in llamas [34,56,95,96], which are far from the high burdens which may be reached in confinement situations of domesticated animals [99,100]. Low burdens in llamas in the open field may be linked to their peculiar defecation behavior. These animals deposit their fecal excretions (fecal particles of llama are commonly called llama beans because of their aspect) in dung piles (Figure 6A–C). Although there may usually be several dung piles within any one field or pasture, it is evident that llamas are extremely hygienic as compared with sheep, goats, cattle, and equines. These dung piles reduce the spreading capacity of the fecal pathogens, opposite to the other livestock species which defecate everywhere and thus contribute to a wider spread of the parasites. Interestingly, a contrary evolutionary trend appears to be followed by the tick species *Amblyomma parvitarsum* (Acari: Ixodidae), which has adapted to this particular defecation behavior and waits in these dung piles to facilitate its access to the llamas (Figure 7F,G) [110]. Moreover, llamas always make these dung piles far away from freshwater collections (Figure 6B), which prevents lymnaeid snail infection by eggs shed by this camelid. Such lower *F. hepatica* intensity rates in llamas do not suggest fascioliasis to induce high pathogenicity pictures shortly leading to death in the open field.

## 5. Concluding Remarks

The results of the present experimental studies demonstrate that the llama is a definitive host species in which *F. hepatica* is able to close its complete life cycle. However, the follow-up of the llama isolate, by means of a highly efficient lymnaeid vector species in altitude areas such as *G. truncatula*, indicates that *F. hepatica* does not reach in this camelid species the level of adaptation this parasite shows in sheep and cattle in Andean altitude areas as in the Northern Bolivian Altiplano.

In the llama isolate, the egg embryonation showed a lack of viability in many eggs and an overall pronouncedly less successful development than in the sheep and cattle isolates. Moreover, results have shown shorter prepatent and patent periods leading to a markedly lower cercarial production capacity, a shorter cercarial shedding chronobiology with only one delayed acrophase, and a higher negative impact on snail survival.

Additionally, the usually low infection intensities of llamas and consequent low daily fecal outputs of liver fluke eggs in Andean endemic areas, together with the peculiar defecation behavior in dung piles of this camelid, should be taken into account.

All in all, the results demonstrate that the llama should not be considered an important definitive host when compared to sheep and cattle in Andean endemic areas [24]. The quantitatively lower contribution of the llama to the transmission of *F. hepatica* and the peculiar defecating behavior in dung piles always far from freshwater collections suggest that there are sufficient reasons as to consider the reservoir role of the llama as negligible and, therefore, no priority within control measures in endemic areas. In the Northern Bolivian Altiplano human hyperendemic area, neither llamas nor alpacas should be considered for control measures within a One Health action.

Complete studies similar to the present one should be performed on other local llama isolates to see whether the liver fluke may perhaps have better adapted to the llama in endemic areas of other Andean countries.

**Figure 8:**
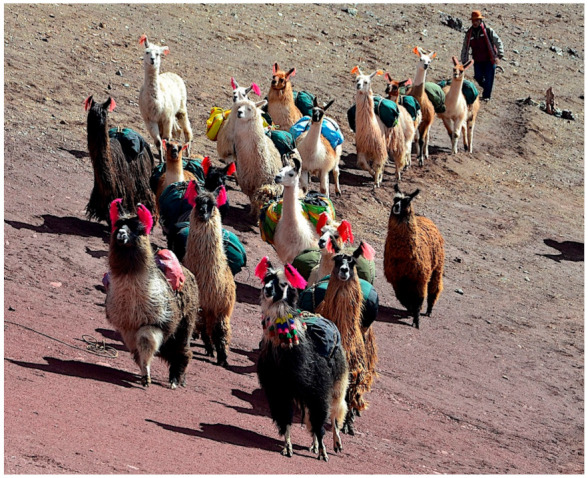
A high altitude Andean caravan of “llamas cargueras” used as pack animals. Note the relatively small packages transported by each animal (compared to the higher weight transporting capacity by donkeys and mules). Slightly modified from a photograph by Andean Lodges, May 2018, freely available from Wikimedia Commons under license of Creative Commons Atribución-CompartirIgual 4.0 Internacional (CC BY-SA 4.0), https://creativecommons.org/licenses/by-sa/4.0/deed.es, accessed on 2 July 2021.

However, it should be considered that llamas may play a disease-spreading role if used as pack animals in rural areas (Figure 7D,E and Figure 8), as already highlighted in the case of donkeys [25] and mules [107], the latter two with a capacity to transport pronouncedly higher weights of goods [111]. Such a fascioliasis-spreading capacity poses a problem for the implementation of a One Health initiative, because pack animals may give rise to movements of the parasite and the vector from one part to another of the zone selected for control intervention, or the introduction of the parasite and/or the vector from outside into that zone. In the case of llamas, pack animals may contribute to the geographical spread of the lymnaeid snails by passively transporting them in mud attached to their hooves.

## Figures and Tables

**Table 1:** Experimental infections of *Galba truncatula* lymnaeid snails from Ancocagua, Northern Bolivian Altiplano human hyperendemic area, with *Fasciola hepatica* llama isolate from Quichagua, Argentina, and their comparison with results obtained in the *F. hepatica* sheep and cattle isolates from the Northern Bolivian Altiplano.

Host Isolate	Llama	Sheep *	Cattle *
*F. hepatica* geographical origin	Quichagua, Argentina	Batallas, Bolivia	Batallas, Bolivia
Lymnaeid geographical origin	Ancocagua, Bolivia	Huacullani, Bolivia	Huacullani, Bolivia
Miracidial dose	mono-miracidial	mono-miracidial	mono-miracidial
Temperature (12h day/12h night)	20 °C/20 °C	20 °C/20 °C	20 °C/20 °C
No. lymnaeids infected	45	62	55
No. survivor snails at beginning of shedding (%)	30 (66.7%)	54 (87.1%)	48 (87.3%)
No. shedding snails (%)	10 (33.3%)	28 (51.8%)	12 (25.0%)
Prepatent period in dpi (mean)	37–50 (39.4)	48–92 (55.6)	49–76 (55.5)
Shedding end in dpi (mean)	37–74 (50.6)	52–136 (89.4)	58–135 (101.6)
Shedding length in days (mean)	1–38 (12.2)	1–88 (34.7)	1–85 (47.1)
No. total cercariae shed	513	5542	3672
No. cercariae/snail (mean)	1–180 (51.3)	8–562 (197.9)	8–581 (306.0)
Snail survival after shedding end in days (mean)	1–31 (7.3)	1–132 (24.5)	1–133 (42.3)
Longevity of shedding snails in dpi (mean)	50–76 (57.9)	53–192 (113.8)	76–268 (143.9)
Longevity of non-shedding snails in dpi (mean)	37–122 (70.8)	49–196 (139.1)	31–209 (105.4)

dpi = days post-infection. * Data from Mas-Coma et al. [24].

**Table 2:** Experimental infections of Wistar rats with experimentally obtained metacercariae from the llama isolate from Quichagua, Argentina, and comparison with sheep and cattle isolates from the Northern Bolivian Altiplano human hyperendemic area.

Host Isolate	Llama	Sheep ***		Cattle ***	
*F. hepatica* geographical origin	Quichagua, Argentina	Batallas, Bolivia	Ancocagua, Bolivia	Kallutaca, Bolivia	Batallas, Bolivia
Age of metacercariae	8–11 weeks	1 week	2 weeks	6 weeks	8 weeks
No. metacercariae inoculated per rat	20	20	20	20	20
No. inoculated rats	25	14	23	4	4
No. rats infected (%)	13 (52.0%)	11 (78.6%)	18 (78.3%)	4 (100%)	2 (50.0%)
No. flukes recovered per rat (mean)	1–10 (5.2)	1–8 (3.6)	1–10 (3.7)	1–2 (1.7)	1–2 (1.5)
Intensity *	13.6%	14.3%	14.6%	8.8%	3.7%
Mean % flukes recovered/rat **	26.1%	18.2%	18.6%	8.8%	7.5%

* Intensity = total % of flukes recovered = (total No. of flukes recovered/total No. of metacercariae administered in all rats) × 100. ** Mean % flukes recovered/rat = Mean % of flukes recovered per infected rat = (flukes recovered/metacercariae administered per infected rat) × 100. *** Data from Mas-Coma et al. [24].

**Table 3:** Comparison of egg measurements (**A**) and egg shedding (**B**) between the *Fasciola hepatica* llama isolate from Argentina and the sheep and cattle isolates from the Northern Bolivian Altiplano human hyperendemic area.

Host			Llama		Sheep		Cattle	
(A) Egg Measurements								
Eggs	Fecal Samples (*n* = 36)		Bile Samples (*n* = 37)		Fecal Samples (*n* = 104)		Fecal Samples (*n* = 168)	
**Measure-ments (µm)**	**Range**	**Mean ± SD**	**Range**	**Mean ± SD**	**Range**	**Mean ± SD**	**Range**	**Mean ± SD**
EL	103.8–139.9	124.2 ± 9.1	105.7–146.7	120.9 ± 9.2	114.8–151.2	130.8 ± 7.1	105.3–155.9	132.0 ± 10.5
EW	57.6–74.5	66.6 ± 3.5	54.9–76.3	65.5 ± 5.4	65.5–81.4	72.6 ± 3.9	61.7–82.5	71.1 ± 4.4
EPe	284.3–350.6	321.8 ± 17.6	277.4–363.2	314.7 ± 19.5	294.2–368.2	327.6 ± 15.0	270.6–422.9	340.0 ± 33.4
EA	5022.4– 7269.5	6399.9 ± 625.1	4761.5–7570.7	6108.8 ± 728.2	5998.2–8608.4	7238.0 ± 532.8	5286.5–9676.8	7170.2 ± 802.5
EL/EW	1.52–2.13	1.8 ± 0.15	1.5–2.3	1.8 ± 0.2	1.5–2.1	1.8 ± 0.1	1.6–2.3	1.8 ± 0.2
**(B) Egg Shedding**								
Intensity (epg)			1–10		3–241 (a)		1–96 (a)	
Stools/day (kg)			0.7–3.9 (c)		1–3 (b)		15–35 (b)	
No.eggs/animal/day (d)			700–39,000		3000–723,000		15,000–3360,000	

EL: egg length; EW: egg width; EPe: egg perimeter; EA: egg area; EL/EW: egg length/egg width. Measurement values shown as range and mean ± standard deviation (S.D.). (a) = according to Mas-Coma et al. [24]; (b) = according to various sources; (c) = after Anderson [76] and Adam and Adam [77]; (d) = estimations of the range of the number of F. hepatica eggs fecally shed by an animal per day in the Bolivian Altiplano.

## Data Availability

The datasets generated for this study are available on request to the corresponding author.
